# Supplementary material for: Achieving High Expression of Cry in Green Tissues and Negligible Expression in Endosperm Simultaneously via rbcS Gene Fusion Strategy in Rice
Source: Int J Mol Sci. 2023 May 20;24(10):9045. doi: 10.3390/ijms24109045 (PMC10218938; doi:10.3390/ijms24109045)
Supplement: Supplementary file 1 [file ijms-24-09045-s001.zip › Table S1.pdf]

Table S1 Content of Cry1Ab/Cry1Ac in rice seeds by ELISA assay

Line	Brown rice	Endosperm
NT	0.000037±0.000033	0.000053±0.000024
RRC-1	0.014587±0.000565**	0.000693±0.000103**
RRC-2	0.019605±0.000381**	0.000955±0.000168**
RRC-3	0.010037±0.000145**	0.001173±0.000164**
RRC-11	0.025013±0.000420**	0.000464±0.000073**
RRC-12	0.005701±0.000033**	0.000117±0.000009
T51-1	1.040330±0.060377**	0.305228±0.027174**

All dada were measured from 10 randomly sampled plants per test material per replication (3 replications) and were analysed by the Tukey's multiple comparisons test using the GraphPad Prism 8 software package. Values are given as the mean±standard deviation (SD). NT as the negative control; All of the others lines were compared with the NT. * and ** meant significantly different from the control at P <0.05 and P<0.01, respectively.
